# Probiotic Strains from Canine Milk Support Gastrointestinal Health in Weaning Labrador Retriever Puppies

**DOI:** 10.3390/ani16030463

**Published:** 2026-02-02

**Authors:** Leónides Fernández, Claudio Alba, Manuela Pérez, David Díaz-Regañón, Melanie Wergin, Stephan Duller, Juan M. Rodríguez

**Affiliations:** 1 Department of Galenic Pharmacy and Food Technology, Complutense University of Madrid, 28040 Madrid, Spain; leonides@ucm.es (L.F.);; 2 Instituto Pluridisciplinar, Complutense University of Madrid, 28040 Madrid, Spain; c.alba@ucm.es; 3 Department of Nutrition and Food Science, Complutense University of Madrid, 28040 Madrid, Spain; drdiazreganon@ucm.es; 4 Biologische Heilmittel Heel GmbH, 76532 Baden-Baden, Germany; 5 Elementrial Clinical Research, 8010 Graz, Austria; stephan.duller@elementrial.com

**Keywords:** Labrador Retriever puppies, weaning, probiotics, canine milk, *Lacticaseibacillus rhamnosus*, *Lactiplantibacillus plantarum*, gastrointestinal health, short-chain fatty acids, antibiotics

## Abstract

Weaning is a critical developmental stage in puppies often accompanied by gastrointestinal disturbances and empiric antibiotic use. This double-blind, placebo-controlled trial evaluated the safety and efficacy of two canine milk-derived probiotic strains, *Lacticaseibacillus rhamnosus* CECT 30021 and *Lactiplantibacillus plantarum* CECT 30022, in recently weaned Labrador Retriever puppies. Forty-nine healthy puppies were enrolled and allocated to receive a daily capsule containing either strain alone, a combination of both, or placebo for six weeks, followed by a six-week observation period. Probiotic supplementation was well tolerated, and clinical, hematological, and biochemical parameters remained within normal ranges. Compared with placebo, the probiotic-treated puppies experienced fewer gastrointestinal infections and required less antibiotic therapy. Supplementation helped maintain normal fecal consistency, prevented increases in fecal calprotectin and inflammatory cytokines, and produced significantly higher fecal short-chain fatty acid concentrations. Systemically, probiotic intake was associated with lower serum cortisol, IL-8, TNF-α, and cholesterol, and stronger antibody responses to vaccination. Probiotic-supplemented puppies also showed faster weight gain during treatment, and this difference persisted after supplementation ended. Overall, these findings indicate that both strains, administered individually or in combination, safely support gastrointestinal, metabolic, endocrine, and immunological health in Labrador Retriever puppies during weaning and may help reduce the need for antibiotics.

## Simple Summary

Weaning is a challenging time for puppies because they are suddenly separated from their mother and must adjust to solid food. During this period, many puppies are at increased risk of digestive problems, which often lead to veterinary visits and antibiotic treatment. In this study, we tested two probiotic strains naturally found in canine milk—*Lacticaseibacillus rhamnosus* CECT 30021 and *Lactiplantibacillus plantarum* CECT 30022—to evaluate their ability to support the health of young Labrador Retriever puppies. The probiotics were safe and well tolerated. Puppies that received them had fewer digestive upsets, required fewer antibiotics, and maintained healthier stool consistency than puppies that did not receive them. They also showed signs of lower stress and inflammation, slightly faster growth, and stronger antibody responses to vaccination. Overall, these probiotics supported gut and general health during the sensitive weaning period.

## 1. Introduction

Infectious gastroenteritis is one of the most frequent veterinary clinical problems encountered in puppies and young dogs [1]. In primary-care populations, acute diarrhea affects roughly 8% of dogs annually [2]. Most episodes are mild and self-limiting, resolving within a few days with dietary modification and supportive care, and current guidelines therefore discourage routine antimicrobial use in uncomplicated cases [3,4,5]. Puppies show the highest prevalence, with reported rates up to 10–25% in the first year of life, particularly around weaning, reflecting immature immunity, dietary transitions, and higher susceptibility to infectious agents, whereas in adult dogs diarrhea more often reflects dietary indiscretion or chronic underlying disease [2]. Nevertheless, diarrhea remains a leading indication for empiric antibiotic prescription in small-animal practice [6,7,8,9]. This pattern persists despite the infrequent use of diagnostic testing [7] and growing evidence that antimicrobials provide no clinically meaningful benefit in dogs with mild or moderate acute diarrhea [5]. Beyond their questionable utility in many such cases, unnecessary antibiotics accelerate the rise and spread of antimicrobial resistance—an urgent One Health concern with major implications for both veterinary and human medicine [10,11,12,13].

Early-life exposure to antibiotics may also interfere with the establishment of the gut microbiota, a process essential for digestive, metabolic, mucosal, and immunological development, particularly around weaning when gastrointestinal and immune functions are still maturing [14,15,16,17,18,19]. However, across all life stages, dysbiosis—especially the loss of butyrate-producing anaerobes—has consistently been associated with acute and chronic gastrointestinal disorders [20].

Probiotics—defined as “live microorganisms that, when administered in adequate amounts, confer a health benefit on the host” [21]—offer a promising, microbiota-friendly avenue to support gastrointestinal health in dogs [22,23]. However, development and testing of canine-adapted probiotic strains remain limited. Many available products rely on strains isolated from humans or other species, and such host mismatch may contribute to the variability and sometimes modest clinical effects reported in canine trials [5]. Increasing evidence suggests that autochthonous, host-specific strains may offer greater colonization potential and more consistent efficacy [22,24,25,26]. In this context, canine-derived probiotics are increasingly used both therapeutically and prophylactically. Prophylactic, longer-term administration of canine-milk-derived probiotics is particularly appealing, since it can support stable intestinal colonization and a more resilient intestinal microbiota during predictable high-risk periods such as weaning or dietary transitions [27].

For practicing veterinarians, the availability of reliable, host-adapted probiotics, especially for prophylactic use around predictable high-risk periods, could help reduce the frequency of diarrhea and thereby limit inappropriate antibiotic use, a relationship supported by clinical trials and current antimicrobial stewardship data [5,9,28].

Against this background, a distinguishing feature of the present research program is the use of two probiotic strains—*Lacticaseibacillus rhamnosus* CECT 30021 and *Lactiplantibacillus plantarum* CECT 30022—originally isolated from canine milk, a biological fluid known to contain bacteria adapted to early-life gut colonization [27]. In a previous randomized trial in German Shepherd and Yorkshire Terrier puppies, these strains significantly reduced gastrointestinal infections and antibiotic use, increased fecal short-chain fatty acid (SCFA) concentrations, strengthened the abundance of beneficial taxa, and supported healthier metabolic profiles [29]. These encouraging results suggested that canine-milk-derived strains may provide biologically relevant benefits during weaning, yet also highlighted the need for confirmatory evidence across different breeds and environments. Such replication is essential because probiotic responses can vary markedly with the strain, host breed and age, study design, and physiological state of the animal [30,31,32].

The present double-blind, placebo-controlled trial was therefore designed to extend previous findings by evaluating these same two canine-milk-derived strains in recently weaned Labrador Retriever puppies. The overall aim was to generate robust, confirmatory data on both safety and efficacy. More specifically, this study sought to (1) verify the safety and tolerability of *L. rhamnosus* CECT 30021 and *L. plantarum* CECT 30022 when administered individually or in combination once daily over six weeks to very young puppies, and (2) assess their potential benefits on gastrointestinal health, fecal and systemic inflammatory markers, metabolic and endocrine parameters, and vaccine antibody responses—factors of direct clinical relevance for veterinarians managing puppies during the vulnerable post-weaning period. By focusing on autochthonous strains and testing them across multiple breeds, this work contributes to a more evidence-based use of probiotics in canine medicine and provides essential information for their evaluation as zootechnical additives under current European Food Safety Authority (EFSA) criteria.

## 2. Materials and Methods

### 2.1. Test Items

The probiotic strains *Lacticaseibacillus rhamnosus* CECT 30021 and *Lactiplantibacillus plantarum* CECT 30022, originally isolated from canine milk and taxonomically identified by 16S rRNA gene sequencing [29], were obtained from Heel GmbH (Baden-Baden, Germany).

Probiotic capsules contained $1\times 10^{9}$ CFU of either *L. rhamnosus* CECT 30021 (HP1 group), *L. plantarum* CECT 30022 (HP2 group), or a combination of both strains (HP1/2 group), with food-grade maltodextrin (Acofarma, Terrassa, Spain) as excipient. Placebo capsules contained only excipient and were indistinguishable in appearance from the probiotic formulations.

### 2.2. Study Design and Treatment Protocol

A prospective, double-blind, placebo-controlled trial with a parallel-group design was conducted to assess the effects of probiotic supplementation on gastrointestinal health in recently weaned Labrador Retriever puppies. Four treatment strategies were compared: HP1 (receiving *L. rhamnosus*), HP2 (receiving *L. plantarum*), HP1/2 (receiving a combination of *L. rhamnosus* and *L. plantarum*), and placebo.

Puppies received one capsule daily, mixed with a small portion of food to ensure ingestion before the remainder of the meal was offered. Treatment lasted for six weeks and was followed by a six-week observation period. Recruitment was performed on the same day or the day after weaning to ensure initiation of supplementation immediately after separation from the dam.

### 2.3. Participants, Housing, and Group Allocation

Recruitment took place between March 2023 and March 2024 at a Labrador Retriever breeding center in Madrid, Spain. Puppies were included if they were healthy, as confirmed by veterinary examination, 5±1 weeks old, and either still nursing or weaned no more than one day before study start. Exclusion criteria included previous probiotic administration (in dam or puppy), evidence of systemic or local inflammation, abnormal hematological parameters, temperature < 35.5 °C or >39.5 °C, heart rate > min^−1^, packed cell volume > 58%, white blood cell count < 5 × 10^9^ cells/L or >20 × 10^9^ cells/L, dehydration, poor body condition or low body weight for age, antibiotic treatment within the past two weeks, positive results for canine parvovirus (CPV) in the puppy or littermates, or insufficient blood volume for baseline analyses.

Puppies were housed under standard breeder husbandry conditions and fed a commercial starter diet (Arion Titanium Starter; Arion España, Tres Cantos, Madrid, Spain). All received routine antiparasitic treatments with praziquantel, pyrantel embonate, febantel, and fenbendazole at weight-adjusted doses, and were vaccinated against CPV, canine distemper virus (CDV), infectious canine hepatitis, and leptospirosis at the appropriate ages, following the WSAVA guidelines.

Eligible puppies were allocated to four treatment groups (placebo, HP1, HP2, or HP1/2) using a balanced allocation schedule ensuring equal sex distribution (6 males and 6 females per group). Assignment codes were sequentially numbered and concealed from investigators, breeders, and laboratory staff, who remained blinded to treatment identity throughout the study.

Sample size was determined to detect a one–standard deviation difference in fecal SCFA concentrations between groups (80% power, one-tailed α=0.05). This effect size was based on a previous canine study using the same probiotic strains, in which statistically significant increases in fecal SCFA concentrations smaller than one standard deviation were observed across different breeds [29]. The resulting target sample size was 11 puppies per group. To accommodate a possible 10% dropout rate, 12 puppies were planned per group (48 in total).

Of the 62 puppies screened between March 2023 and March 2024, 13 were excluded due to low body weight (n=6), co-litter infection with CPV (n=5), or insufficient initial blood volume (n=2). The remaining 49 puppies were enrolled in the study. Initially, 48 puppies were assigned to the four study groups (12 per group) to ensure balanced group sizes and sex distribution. During the study, one placebo-treated puppy died of gastroenteritis unresponsive to antibiotic and fluid therapy. To preserve balanced group sizes and sex distribution, an additional sex-matched puppy was subsequently enrolled. Data collected from the animal that died were included up to the time of death. A total of 48 puppies completed the study and were included in the final Day 42 analyses.

### 2.4. Ethical Approval

The study was approved by the Ethics Committee of the Complutense University of Madrid (Reference NP0121012021-2021). All procedures complied with EU Directive 2010/63/EU on the protection of animals used for scientific purposes and Commission Recommendation 2007/526/CE on housing and care. Animal health and welfare were monitored throughout the trial. No procedures required sedation or analgesia beyond standard clinical practice.

### 2.5. Veterinary Clinical Examinations and Sampling

Immediately before recruitment and every seven days thereafter, all puppies underwent a veterinary clinical examination to record general health status. Body weight was measured at the start, every two weeks during the six-week supplementation period (Days 0, 14, 28, and 42), and again after the six-week observation period (Day 84).

Blood and fecal samples were collected at baseline (Day 0) and at the end of the supplementation period (Day 42). Blood was drawn from the jugular vein into tubes with and without EDTA (Aquisel, Abrera, Spain) to obtain whole blood, plasma, and serum fractions. Serum and plasma samples were aliquoted as appropriate and stored at −80 °C until analysis. Fecal samples were collected immediately after defecation and frozen at −80 °C until analysis.

### 2.6. Blood and Serum Analyses

All hematological and standard biochemical analyses were performed by the diagnostic laboratory of the Faculty of Veterinary Medicine, Complutense University of Madrid (Spain), using standard validated methods.

Complete blood counts were determined using an automated hematology analyzer (ProCyte Dx Hematology Analyzer; IDEXX Laboratories, Westbrook, ME, USA). Hematological parameters included red blood cell (RBC) count, hemoglobin, hematocrit, mean corpuscular volume (MCV), mean corpuscular hemoglobin (MCH), mean corpuscular hemoglobin concentration (MCHC), red blood cell distribution width (RDW), hemoglobin distribution width (HDW), and percentage of reticulocytes. White blood cell (WBC) parameters comprised total leukocytes, neutrophils, lymphocytes, monocytes, eosinophils, and basophils. Platelet count, mean platelet volume (MPV), and platelet distribution width (PDW) were also recorded.

Serum biochemical analyses included glucose, blood urea nitrogen (BUN), uric acid, creatinine, total and conjugated bilirubin, total cholesterol, electrolytes (potassium, chloride, sodium, calcium, phosphorus), and enzymatic activities of alkaline phosphatase (ALP), aspartate aminotransferase (AST), alanine aminotransferase (ALT), gamma-glutamyl transferase (GGT), total lactate dehydrogenase (LDH), creatine phosphokinase (CPK), amylase, and lipase. Total protein, albumin, and globulin concentrations were measured, and the albumin:globulin ratio (A/G) was calculated accordingly.

Canine pancreatic-specific lipase (cPLI) was measured by Laboratorio de Análisis Veterinarios (LAV, Arturo Soria S.L., Madrid, Spain).

Plasma concentrations of cortisol were quantified using a commercial canine-specific ELISA kit (MyBioSource, San Diego, CA, USA). Cytokines IL-8, IL-10, and TNF-α were analyzed in serum by canine-specific ELISA kits (Thermo Fisher Scientific, Waltham, MA, USA). Antibody titers against CPV and CDV were semi-quantitatively determined using the Canine VacciCheck Antibody Test Kit (Biogal Galed Labs, Kibbutz Galed, Israel). All antibody tests were performed on serum collected at Day 42, following vaccination during the supplementation period.

### 2.7. Fecal Analyses

The presence of CPV type 2 was assessed in all fecal samples using a commercial lateral-flow immunochromatographic assay (LiliF Canine Parvovirus Ag Rapid Test Kit; iNtRON Biotechnology, Seongnam, Republic of Korea).

Fecal consistency was evaluated at baseline and Day 42 using the Purina canine fecal scoring system [33], which rates stool texture from 1 (very hard and dry) to 7 (watery, no texture). Scores of 3 were considered normal, while scores < 2 indicated constipation and scores > 3 indicated diarrhea. Fecal dry matter was determined by drying duplicate aliquots at 105 °C for 24 h until constant weight; the results were expressed as percentage dry matter.

Fecal SCFAs (acetate, propionate, butyrate) were quantified by gas chromatography–mass spectrometry (GC–MS) as described previously [34], and previously validated for canine feces [29,35]. Fecal calprotectin concentrations were determined by ELISA (Canine Calprotectin ELISA Kit; MyBioSource, San Diego, CA, USA). Cytokines IL-6, IL-8, IL-10, and TNF-α in feces were measured using canine-specific ELISA kits (Thermo Fisher Scientific, Waltham, MA, USA).

### 2.8. Statistical Analyses

All statistical analyses were performed using *Jamovi* software (version 2.7; The Jamovi Project, Sydney, Australia), which applies R-based statistical computation. Descriptive statistics were used to summarize data distributions for all hematological, biochemical, fecal, and immunological variables. The results are presented as medians and ranges (minimum–maximum) in the tables, or as individual data points with group medians in the figures.

For between-group comparisons at Day 42, data were analyzed using the Kruskal–Wallis test followed by Dunn’s pairwise post hoc tests versus placebo. Bonferroni correction was applied to adjust *p*-values for multiple pairwise comparisons per parameter. For overall group comparisons, effect sizes were expressed as ($\varepsilon^{2}$) for Kruskal–Wallis tests, whereas effect sizes for Dunn’s post hoc pairwise comparisons were reported as correlation coefficients (*r*).

For parameters in which values below the lower limit of detection (LLOD) occurred in the majority of samples, no inferential statistical analyses were performed. The results were reported descriptively as counts above the LLOD.

Safety outcomes and adverse events were evaluated descriptively.

For the analysis of body weight during the supplementation period (Days 0–42), a linear mixed-effects model (LMM) was applied using the GAMLj3 module in Jamovi, with treatment group and sex as fixed factors, puppy as a random factor, and baseline weight as a covariate. Weights at follow-up (Day 84) were analyzed by analysis of covariance (ANCOVA) with baseline body weight as covariate.

A two-sided *p*-value < 0.05 was considered statistically significant.

## 3. Results

### 3.1. Number of Animals and Baseline Characteristics

A total of 49 healthy Labrador Retriever puppies were enrolled in the study and received at least one dose of either placebo or probiotic preparation (HP1, HP2, or HP1/2), including one animal enrolled to replace a puppy that died during the study. Numbers of animals per treatment group, baseline age, and sex distribution were similar and evenly balanced between groups (Table 1). Mean body weight at baseline was numerically higher in the placebo group compared with the probiotic groups, but all puppies’ weights were within the normal range for this breed and age. No differences in age or weight were observed between male and female puppies.

One puppy in the placebo group died prematurely. All final Day 42 analyses were therefore conducted on 48 puppies (12 per group; 6 males and 6 females).

### 3.2. Adverse Events and Mortality

A total of 6 adverse events (AEs) were recorded across 6 individual animals, all classified as gastroenteritis (diarrhea; Table 2). Among the 13 puppies receiving placebo, 5 (38%) developed gastroenteritis requiring antibiotic treatment. One of these cases was unresponsive to combined antibiotic therapy (metronidazole and cotrimoxazole) and fluid therapy, and the puppy died on Day 35 after treatment initiation.

Only one case of diarrhea occurred in the HP2 group. This episode was associated with the accidental ingestion of ivy seeds and resolved completely with fluid therapy, without the need for antibiotics. No AEs were observed in HP1 or HP1/2 groups.

Hematological (Table A2) and blood biochemistry (Table A3) parameters measured at the beginning (Day 0) and end (Day 42) of the treatment period were within normal reference ranges for this breed and age. Minor variations were attributed to physiological changes associated with growth. No clinically relevant or treatment-related abnormalities were detected in any of the probiotic groups. cPLI was below the detection limit (<40 µg/L) in all samples, indicating no evidence of pancreatic inflammation. Likewise, the analysis of serum IL-8, IL-10, and TNF-α did not indicate any relevant systemic inflammatory response (Table A4).

No AEs attributable to probiotic administration were documented throughout the six-week treatment or the six-week follow-up period. None of the puppies receiving probiotic preparations died prematurely, confirming the safety and tolerability of all probiotic treatments.

### 3.3. Effect of Probiotic Supplementation on Body Weight Gain

Body weight was recorded every two weeks from the start of the study, when the puppies were 5 ± 1 weeks old, until the end of the six-week treatment period at 11 weeks of age. Weights at 7, 9, and 11 weeks were analyzed using a LMM with treatment group and sex as fixed factors and puppy as a random factor. Baseline weight was included as a covariate to adjust for initial differences. Estimated marginal means (EMMeans) with 95% confidence intervals were compared among treatment groups at each time point.

**Figure 1:**
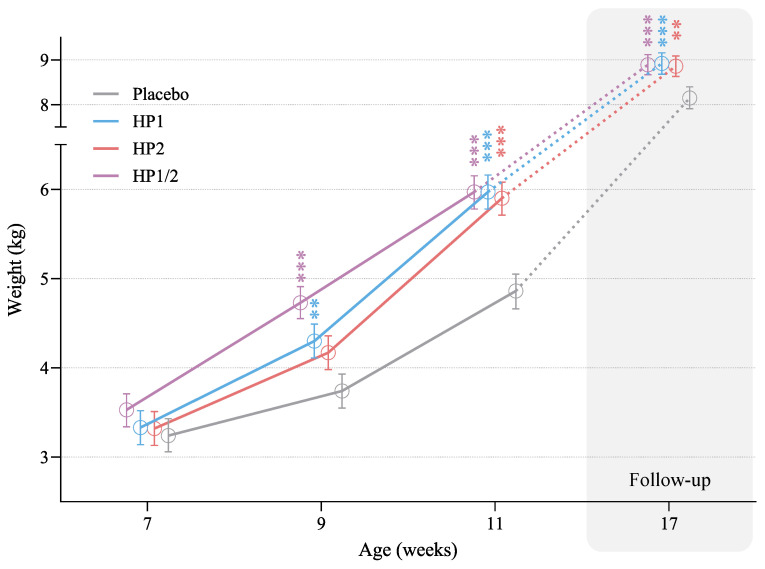
Body weight gain in Labrador Retriever puppies receiving placebo or probiotic supplementation (HP1, HP2, HP1/2) during the six-week treatment period (5–11 weeks of age) and at follow-up (17 weeks). Probiotic supplementation resulted in significantly faster body weight gain during the treatment phase, and this difference persisted at follow-up. Values represent estimated marginal means (±95% confidence intervals). Significant differences versus placebo at each time point are indicated (** *p* < 0.01; *** *p* < 0.001; LMM for the treatment phase, ANCOVA for the follow-up).

Puppies that received probiotic supplementation gained weight faster than those given placebo (Table 3, Figure 1). At 9 weeks of age—about four weeks after supplementation began—EMMeans were significantly higher in the HP1 (*p* = 0.008) and HP1/2 (*p* < 0.001) groups compared with placebo. By the end of the treatment period, all three probiotic groups showed significantly greater body weights than the placebo group (*p* < 0.001 for all comparisons), with mean differences of roughly one kilogram.

To assess whether this difference persisted after treatment, body weight was measured again at 17 weeks of age, corresponding to six weeks of follow-up. An ANCOVA including treatment and sex as factors, with baseline body weight as a covariate, confirmed that the difference in weight gain was maintained: puppies that had received probiotics remained about 0.7 kg heavier than those in the placebo group (*p* < 0.01).

Sex had no significant effect in either model, and there was no interaction between sex and treatment. Despite the differences among groups, body weights for all puppies remained within the normal range for Labrador Retrievers at every measurement time.

### 3.4. Effect of Probiotic Supplementation on Fecal Characteristics

At baseline, all fecal samples showed normal consistency. Purina fecal scores were highly consistent, ranging from *3* in 39 samples to *4* in 10 samples, indicating well-formed stools. Mean dry matter content ranged from 27.1 to 29.9% across all groups (Table A1). SCFAs—acetate, propionate, and butyrate—were detected in all samples at low baseline concentrations. Fecal calprotectin averaged about 0.12 µg/g. None of the inflammatory cytokines (IL-6, IL-8, IL-10, TNF-α) were detectable in any baseline sample, consistent with a healthy baseline status of the puppies before treatment.

**Figure 2:**
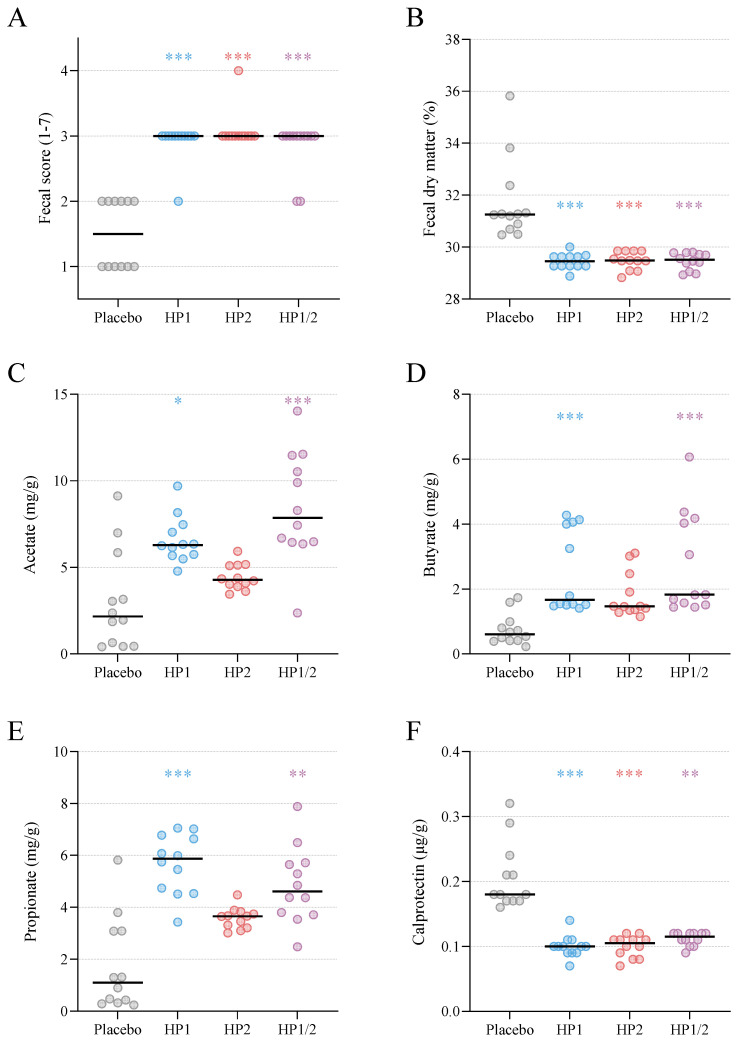
Fecal characteristics and biochemical parameters at Day 42 in Labrador Retriever puppies receiving placebo or probiotic supplementation: (**A**) Individual fecal scores (1–7; normal = 3). The placebo group showed scores of 1–2, indicating dry, hard stools, whereas most probiotic-treated puppies had normal scores of 3. (**B**) Fecal dry-matter content was significantly lower in all probiotic groups than in the placebo group (*p* < 0.001 for all; Kruskal–Wallis test with Dunn’s post-test). (**C**) Acetate, (**D**) butyrate, (**E**) propionate, and (**F**) calprotectin concentrations in fecal samples. Probiotic supplementation increased SCFA concentrations—particularly acetate, butyrate, and propionate in HP1 and HP1/2 groups—and prevented the rise in fecal calprotectin observed in placebo animals. Significant differences compared with placebo were determined by Kruskal–Wallis tests followed by Dunn’s post hoc tests. Data points represent individual values; horizontal bars indicate group medians. * *p* < 0.05, ** *p* < 0.01, *** *p* < 0.001.

After six weeks of supplementation on Day 42, fecal samples were re-evaluated for consistency and biochemical parameters. All placebo-treated puppies showed a shift toward harder and drier stools, with a fecal score of *2* in 6 animals and a fecal score of *1* in 6 animals. Accordingly, fecal dry matter content exceeded 30% in all placebo samples. In contrast, puppies supplemented with any of the probiotic preparations maintained normal stool consistency: most had a fecal score of *3*, with only isolated cases scoring *2* (1 in HP1, none in HP2, and 2 in HP1/2). Dry matter content in the probiotic groups remained below 30%. A Kruskal–Wallis test comparing fecal scores and fecal dry matter among the four groups at Day 42 showed significantly healthier values in all probiotic groups than in placebo (*p* < 0.001 for each) (Figure 2A,B and Table A5).

Analysis of SCFA concentrations at Day 42 revealed marked increases in puppies receiving probiotics (Figure 2C–E). Acetate, butyrate, and propionate levels were all significantly higher in the HP1 and HP1/2 groups compared with placebo (*p* < 0.01 or *p* < 0.001; Kruskal–Wallis test with Dunn’s post-test). In the HP2 group, median concentrations of all three SCFAs were numerically higher than in the placebo group; however, these moderate increases did not reach statistical significance (Table A5).

Fecal calprotectin concentrations approximately doubled in the placebo group but remained stable in all probiotic groups. At Day 42, median calprotectin levels were significantly lower in all probiotic groups than in placebo (Figure 2F; *p* < 0.01 or *p* < 0.001), indicating reduced intestinal inflammation. Consistent with this attenuated mucosal inflammatory response, fecal inflammatory cytokines IL-6 and IL-8 at concentrations above the LLOD were detected almost exclusively in the placebo group (7 and 11 of the 12 animals, respectively), whereas only one probiotic-treated puppy (1/36; HP2 group) had concentrations above the LLOD (Table A1).

All fecal samples tested negative for CPV at both time points, indicating no evidence that the observed gastrointestinal changes were associated with CPV infection.

### 3.5. Systemic and Immunometabolic Effects of Probiotic Supplementation

At baseline, serum concentrations of cortisol, IL-8, TNF-α, and IL-10 were low in all groups, with no indication of systemic inflammation or stress (Table A4). These results confirmed that all puppies were clinically healthy at study initiation.

**Figure 3:**
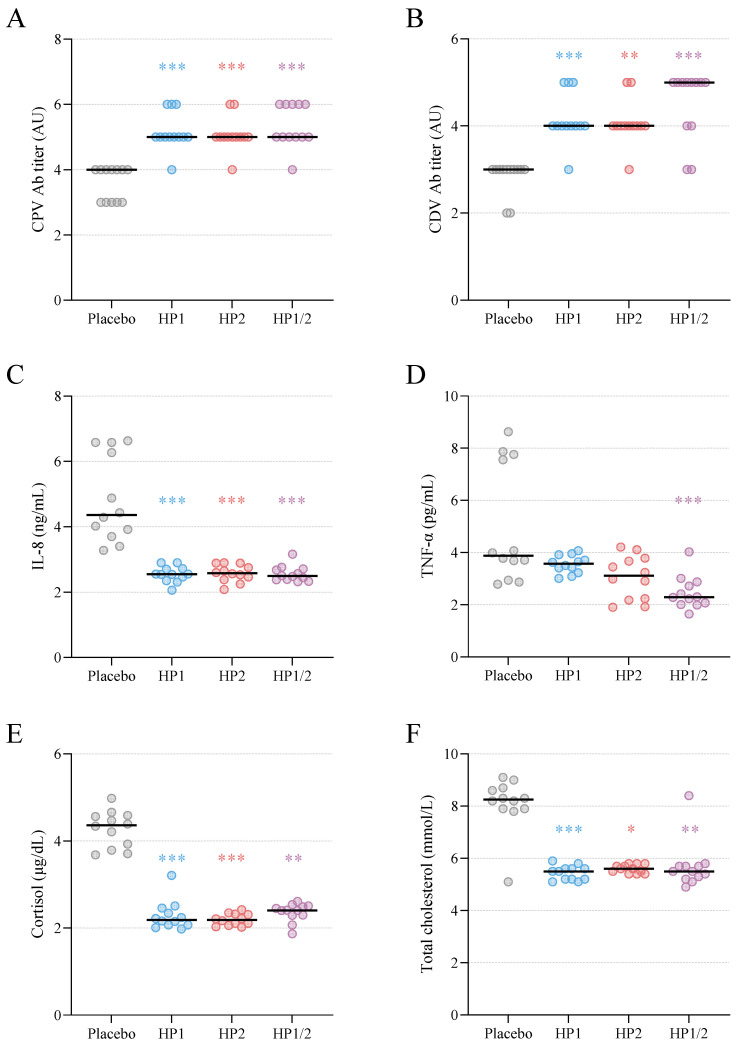
Systemic immunological, endocrine, and metabolic parameters at Day 42 in Labrador Retriever puppies receiving placebo or probiotic supplementation. Probiotic supplementation led to higher vaccine antibody responses against (**A**) canine parvovirus (CPV) and (**B**) canine distemper virus (CDV). In the probiotic groups, circulating levels of IL-8 (**C**), TNF-α (**D**), cortisol (**E**), and total cholesterol (**F**) were reduced compared with placebo. Significant differences versus placebo were determined by Kruskal–Wallis tests followed by Dunn’s post hoc tests. Data points represent individual values; horizontal bars indicate group medians. * *p* < 0.05, ** *p* < 0.01, *** *p* < 0.001.

After six weeks of supplementation on Day 42, clear group differences were observed in circulating biomarkers related to stress and inflammation (Figure 3). Serum cortisol concentrations (Figure 3E) were markedly higher in the placebo group, whereas all probiotic groups showed substantially lower values. Differences between the placebo and each probiotic group were statistically significant (*p* < 0.01 or *p* < 0.001 for all comparisons; Table A4 and Table A5). Similarly, serum IL-8 concentrations (Figure 3C) were significantly lower in all probiotic groups than in the placebo group at Day 42, even though IL-8 levels in the placebo animals did not reach values indicative of severe systemic inflammation. TNF-α concentrations (Figure 3D) were also lower in probiotic-treated puppies, reaching statistical significance only in the HP1/2 group. IL-10 remained mostly below the limit of detection; however, it was measurable in 4 of 12 placebo samples (33%) and in 1 of 12 HP2 samples (8%).

All puppies developed protective antibody titers following vaccination against CPV and CDV administered during the treatment period. At Day 42, antibody titers were higher in all probiotic groups than in the placebo group (Figure 3A,B; Table A4 and Table A5), suggesting an enhanced systemic immune response to vaccination.

Hematological and biochemical profiles were within normal reference ranges for all groups at both sampling times (Table A2 and Table A3). Minor between-group variations were observed for some individual parameters (Table A5), but none indicated pathological changes. Notably, total cholesterol concentrations (Figure 3F) were markedly elevated in placebo animals at Day 42, whereas levels remained significantly lower and comparable among all probiotic groups.

## 4. Discussion

In recent years, increasing attention has focused on developing probiotic strains specifically adapted to canine physiology [22,36,37]. However, clinical evidence in dogs remains limited and often variable, reflecting differences in strain selection, formulation, study design, and host-related factors [5,38]. This variability underscores the importance of conducting confirmatory studies in diverse canine populations. The present trial evaluated the safety and efficacy of once-daily treatment over six weeks with *Lacticaseibacillus rhamnosus* CECT 30021 and *Lactiplantibacillus plantarum* CECT 30022—two strains originally isolated from canine milk [29]—in recently weaned Labrador Retriever puppies. Supplementation with these strains was safe and conferred multiple benefits, confirming and extending previous findings in German Shepherd and Yorkshire Terrier puppies [29]. In Labrador puppies, the probiotics improved gastrointestinal stability, reduced diarrhea and antibiotic use, enhanced SCFA production, and attenuated stress- and inflammation-associated responses, collectively supporting a healthier post-weaning transition.

### 4.1. Safety and Tolerability

No AEs related to probiotic intake were reported by either the supervising veterinary clinician or the breeders throughout the six-week supplementation period and subsequent six-week follow-up. All clinical, hematological, and biochemical measurements remained within the normal reference ranges for the breed at all time points. Although safety data in canine probiotic trials remain relatively scarce, available studies consistently indicate that probiotic administration is well tolerated in dogs [5,38]. The present results, therefore, provide additional evidence that *L. rhamnosus* CECT 30021 and *L. plantarum* CECT 30022 can be safely administered to weaned puppies. Because adults are generally more physiologically robust than puppies, these findings also suggest a favorable safety margin for adult dogs, although this will need to be confirmed in dedicated studies.

### 4.2. Growth Performance

In the current Labrador trial, puppies receiving probiotic supplementation gained weight faster than those in the placebo group. This outcome, consistent with previous findings in other breeds [29], reflects an appropriate growth trajectory rather than excessive growth. Early post-weaning weight gain is widely recognized as an indicator of adequate development and supports a successful transition to permanent homes [39]. Similar improvements in overall health have been observed in other canine probiotic trials [40].

The greater growth performance observed here may result from improved nutrient assimilation and gastrointestinal efficiency, supported by SCFA-mediated metabolic effects. Comparable findings in young dogs and livestock indicate that lactic acid bacteria can strengthen intestinal barrier integrity and fermentation efficiency, thereby improving nutrient utilization and energy yield [40,41,42].

Interestingly, probiotics may also facilitate weight loss in obese dogs [43,44]. Elevated acetic and butyric acid production has been associated both with greater weight gain in growing puppies [29] and with enhanced weight loss in obese adults [40], suggesting that probiotics modulate energy metabolism in a state-dependent manner. However, such effects are not consistently observed and may vary with the probiotic strain, treatment duration, breed, or physiological condition of the host; for instance, no weight differences were reported in juvenile Beagles supplemented with *Limosilactobacillus reuteri* for 21 days [45].

### 4.3. Treatment Duration and Time to Effect

The temporal pattern of body-weight gain provides insight into how long probiotic supplementation must be continued before benefits emerge and how long they persist. Although probiotic-supplemented puppies were numerically heavier at the first follow-up, statistically significant differences versus placebo appeared only after about four weeks of supplementation (9 weeks of age), became more pronounced by the end of the six-week treatment period (11 weeks; Figure 1), and then gradually attenuated during the six-week post-treatment follow-up while remaining detectable at 17 weeks. A similar pattern was seen in a previous trial, where the same canine-milk-derived strains produced significant gastrointestinal and metabolic benefits only after the first four weeks of supplementation [29]. Most other efficacy readouts in the present study (fecal SCFAs, calprotectin, cytokines, serum biomarkers) were measured only at baseline and Day 42, but their concordant improvement at the end of treatment likewise suggests that several effects required sustained daily administration over multiple weeks. Together, these findings support the use of canine-milk-derived probiotics primarily as longer-term, prophylactic interventions around predictable high-risk periods such as weaning, rather than as very short courses intended for rapid, acute therapeutic effects, which in dogs have generally produced only modest clinical benefits [5].

### 4.4. Gastrointestinal Health

Probiotic administration was associated with a markedly lower frequency of gastroenteritis episodes, in line with previous findings in other breeds [29]. In the current trial, 5 of 13 puppies (38%) in the placebo group developed gastroenteritis requiring antibiotic treatment within the six-week post-weaning period. Puppies are inherently prone to infectious gastroenteritis [1], and such episodes remain among the leading indications for empiric antibiotic prescription in primary veterinary practice [6,7]. This is concerning given the contribution of unnecessary antibiotic use to increasing antimicrobial resistance in both pets and humans [10,11], the potential for multidrug-resistant infections [12], and the risk of disrupting early-life microbiota development—an essential determinant of metabolic, immunological, and mucosal maturation [14,20]. Thus, the gastroenteritis cases observed in the placebo group are unsurprising and—as they required antibiotic therapy—highlight the importance of microbiota-friendly preventive strategies in recently weaned puppies.

In contrast, only 1 of the 36 puppies (3%) receiving either strain alone or the combination developed gastroenteritis, and this case was attributable to accidental ingestion of ivy seeds; it resolved with fluid therapy without the need for antibiotics. Probiotic supplementation therefore produced a pronounced reduction in gastroenteritis incidence and antibiotic use compared with placebo. Similar benefits have been reported for formulations containing *L. rhamnosus* and *L. plantarum* in dogs receiving NSAIDs [46]. Collectively, current evidence suggests that probiotics are particularly effective in preventing diarrhea in dogs [28,47], with meta-analyses indicating stronger preventive than therapeutic effects—especially in vulnerable populations such as recently weaned puppies [5,48].

### 4.5. Fecal Characteristics and Intestinal Inflammation

By the end of the supplementation period, all placebo-treated puppies developed unusually firm and dry stools (scores 1–2 with dry matter >30%), whereas probiotic-supplemented puppies largely retained a normal fecal consistency (predominantly score 3), indicating preservation of healthy stool quality under probiotic treatment. Such differences denote improved digestive function in probiotic-treated animals and are consistent with previous work demonstrating that prebiotic fiber blends enhance stool characteristics in puppies [49].

Probiotic supplementation—particularly *L. rhamnosus* and the combined formulation—also led to increased fecal concentrations of acetate, propionate, and butyrate, reproducing the pattern observed in other breeds [29]. SCFAs are central mediators of host–microbiome crosstalk, influencing epithelial energy metabolism, barrier integrity, and immune and neuroendocrine signaling [42,50,51]. Their elevation in probiotic groups is consistent with increased lactic acid production by the administered strains, which may promote the growth of beneficial strict anaerobes and suppress enteropathobionts. Conversely, reduced SCFA concentrations have been associated with gut dysbiosis and gastrointestinal disease in dogs [20,52].

SCFAs have been described to strengthen intestinal barrier integrity by regulating mucin and tight-junction expression and by dampening inflammatory cascades [42,53,54]. This mechanistic link aligns with the observation that fecal calprotectin—a biomarker of intestinal inflammation [55]—was increased in placebo animals and remained stable in probiotic-treated puppies. Elevated fecal calprotectin is characteristic of gastrointestinal disorders in dogs [56,57,58], and similar reductions have been reported in weaning puppies receiving bovine colostrum combined with probiotics [59].

In line with this anti-inflammatory profile, fecal IL-6 and IL-8 were found predominantly in placebo puppies but were largely undetectable in probiotic groups, consistent with reduced mucosal inflammation. Mechanistic support for this effect comes from studies showing that canine-derived strains can suppress lipopolysaccharide (LPS)-induced IL-8 secretion in enterocytes [25], and that probiotics can reduce IL-8 production in gingival epithelial cells exposed to periodontopathogens [60]. Lactic acid bacteria isolated from canine milk reduced fecal TNF-α in mice [61]. In the present study, fecal TNF-α as well as IL-10 were below the detection limit across groups, consistent with a generally low-inflammatory gut environment during early life.

### 4.6. Systemic Immunometabolic and Neuroendocrine Responses

Probiotic supplementation also led to favorable changes in systemic inflammatory and immune-related biomarkers. Serum IL-8 concentrations were markedly higher in placebo puppies, whereas values remained low across all probiotic groups, consistent with the attenuated mucosal inflammation observed in fecal cytokine profiles. TNF-α was likewise reduced, particularly in the combined HP1/2 group. These findings align with experimental evidence showing that canine-derived lactobacilli can downregulate pro-inflammatory cytokine production in epithelial and immune cells [25,60], and that multistrain probiotics can lower systemic TNF-α in dogs [40].

Probiotic intake was also associated with an enhanced vaccine-specific immune response, as puppies receiving any probiotic formulation developed higher CPV antibody titers than those given placebo. Similar immunopotentiating effects have been reported in humans, where probiotics can augment vaccine responses and improve seroconversion [62,63,64].

While all blood chemistry values fell within reference ranges, total cholesterol increased noticeably in placebo puppies but remained stable in all probiotic groups. This protective pattern aligns with evidence from human studies showing cholesterol-lowering effects of probiotics [65] and with similar findings in dogs fed *Enterococcus faecium*–based synbiotics [66].

Systemic endocrine responses were also modulated: At the end of the treatment period, cortisol concentrations were significantly lower in all probiotic groups than in the placebo group. Given that weaning is a period characterized by abrupt social separation and heightened susceptibility to stress, this attenuation of HPA-axis activation is likely meaningful. SCFAs are known to influence gut–brain communication and can dampen stress-induced cortisol responses [67,68], offering a mechanistic link between probiotic-driven metabolic changes and neuroendocrine outcomes. Supporting the relevance of this pathway, a recent study demonstrated that probiotic supplementation for six weeks improved behavior and reduced salivary cortisol in anxious Labrador Retrievers [69]. Although data in dogs remain limited, these findings highlight the potential value of canine-adapted probiotics for mitigating stress reactivity—including in working dogs frequently exposed to challenging environments.

### 4.7. Study Context and Limitations

This study was pre-notified to the European Food Safety Authority (EFSA) for use in the registration process of these strains as zootechnical additives. In this regulatory framework, microbiome sequencing is not required and is in fact discouraged, owing to the absence of standardized criteria for defining a “normal gut microbiome.” Consequently, microbiota profiling was not performed here, although a previous trial with the same strains showed higher abundances of *Lactobacillus* and *Faecalibacterium* DNA in treated puppies [29]. The absence of a direct analysis of the fecal microbiota composition constitutes a major methodological limitation of this study. However, analysis of fecal short-chain fatty acid (SCFA) concentrations was selected to partially address this limitation, since shifts in their levels are commonly considered indirect indicators of treatment-related effects on SCFA-producing microbial populations.

Sampling constraints also imposed practical limitations: because the animals were only a few weeks old, blood collection volumes were kept minimal and performed without sedation to minimize stress, which restricted the number of analytes measured. Fecal samples were obtained individually, either by gentle abdominal massage to stimulate defecation or by collecting a small amount of feces with a sterile swab, ensuring that no cross-contamination occurred. The method inherently yielded the small sample quantities typical for very young puppies. Despite these limitations, the consistency of the results across physiological, biochemical, and inflammatory parameters provides robust support for the observed effects.

## 5. Conclusions

The results of this work support the use of *Lacticaseibacillus rhamnosus* CECT 30021 and *Lactiplantibacillus plantarum* CECT 30022 as probiotics during weaning. Administration of these strains, alone or in combination, reduced gastrointestinal infections, supported growth, increased fecal SCFA concentrations, and beneficially modulated several metabolic, endocrine, and immunological parameters, including cortisol, IL-8, calprotectin, and stronger antibody responses to vaccination. These findings strengthen the evidence base for the safe use of canine-milk-derived probiotic strains to promote gut and systemic health during early life and provide relevant data for their evaluation as zootechnical additives under EFSA guidance.

## Figures and Tables

**Table 1:** Baseline characteristics of Labrador Retriever puppies included in the study.

	Placebo	HP1	HP2	HP1/2
Number of animals	13	12	12	12
Female | Male	7 * | 6	6 | 6	6 | 6	6 | 6
Mean Age (weeks)	4.76	4.70	4.74	4.95
Min–Max	4.57–5.14	4.29–4.86	4.29–5.43	4.57–5.43
Mean Weight (kg)	2.71	2.17	2.20	2.54
Min–Max	2.06–3.26	1.75–2.56	1.62–2.97	2.12–3.02

* Originally, 6 female puppies were enrolled in the placebo group. One puppy died prematurely and was replaced by a sex- and age-matched animal, yielding a total of 7 females in this group.

**Table 2:** Summary of adverse events (AEs) and mortality during the study period.

	Placebo (n=13)	HP1 (*n* = 12)	HP2 (*n* = 12)	HP1/2 (*n* = 12)
Mortality, *n* (%)	1 (8%)	0 (0%)	0 (0%)	0 (0%)
AEs, *n* (%)	5 (38%)	0 (0%)	1 (8%)	0 (0%)
Gastroenteritis *	5 (38%)	0 (0%)	1 (8%)	0 (0%)

* Four cases in the placebo group resolved with antibiotic treatment; one case was unresponsive to therapy and led to death. Case in the HP2 group was related to ingestion of ivy seeds and resolved with fluid therapy.

**Table 3:** Body weight (EMMeans ± SE) during the treatment period (ages 5–11 weeks) and at follow-up (17 weeks).

Age (Weeks)	Treatment	EMMean (SE)	Difference (SE) vs. Placebo	*p*-Value for Difference
7	Placebo	3.24 (0.095)		
	HP1	3.33 (0.096)	0.08 (0.142)	1.000
	HP2	3.32 (0.095)	0.07 (0.141)	1.000
	HP1/2	3.53 (0.093)	0.28 (0.128)	0.546
9	Placebo	3.74 (0.095)		
	HP1	4.30 (0.096)	0.56 (0.142)	0.008
	HP2	4.17 (0.095)	0.43 (0.141)	0.109
	HP1/2	4.73 (0.093)	0.99 (0.128)	<0.001
11	Placebo	4.86 (0.098)		
	HP1	5.97 (0.096)	1.12 (0.144)	<0.001
	HP2	5.90 (0.095)	1.04 (0.143)	<0.001
	HP1/2	5.97 (0.093)	1.11 (0.131)	<0.001
17	Placebo	8.15 (0.123)		
	HP1	8.92 (0.118)	0.77 (0.186)	<0.001
	HP2	8.86 (0.115)	0.71 (0.182)	0.002
	HP1/2	8.89 (0.112)	0.74 (0.156)	<0.001

EMMeans obtained from the LMM (ages 7–11 weeks) and from ANCOVA (17 weeks) adjusted for baseline body weight.

## Data Availability

The raw data supporting the conclusions of this article will be made available by the authors upon request.

## Abbreviations

The following abbreviations are used in this manuscript:

AE	Adverse event
ANCOVA	Analysis of covariance
CDV	Canine distemper virus
CI	Confidence interval
CPV	Canine parvovirus
cPLI	Canine pancreatic specific lipase
EFSA	European Food Safety Authority
HP1	*Lacticaseibacillus rhamnosus* CECT 30021
HP2	*Lactiplantibacillus plantarum* CECT 30022
HP1/2	Combination of *L. rhamnosus* CECT 30021 and *L. plantarum* CECT 30022
IL	Interleukin
LLOD	Lower limit of detection
LMM	Linear mixed model
SCFA	Short-chain fatty acid
SE	Standard error
TNF-α	Tumor necrosis factor alpha

## Appendix A

**Table A1:** Fecal consistency and biochemical characteristics (median and range, min–max) at baseline (Day 0) and after six weeks (Day 42) of placebo or probiotic supplementation.

	Placebo		HP1		HP2		HP1/2	
	Day 0	Day 42	Day 0	Day 42	Day 0	Day 42	Day 0	Day 42
Fecal Score (1–7)	3	1.5	3	3	3	3	3	3
	3–4	1–2	3–4	2–3	3–4	3–4	3–4	2–3
Dry matter (%)	28.59	31.26	27.81	29.45	27.96	29.48	28.11	29.51
	27.34–29.85	30.47–35.82	27.34–29.00	28.88–30.00	27.08–28.33	28.82–29.85	27.11–29.62	28.93–29.80
Butyrate (mg/g)	0.40	0.61	0.39	1.67	0.35	1.46	0.31	1.82
	0.13–0.60	0.23–1.73	0.15–0.51	1.41–4.27	0.21–0.55	1.15–3.11	0.13–0.69	1.44–6.07
Acetate (mg/g)	0.38	2.17	0.32	6.29	0.39	4.28	0.35	7.86
	0.25–5.26	0.41–9.13	0.21–0.38	4.79–9.70	0.21–1.00	3.45–5.93	0.25–1.05	2.37–14.03
Propionate (mg/g)	0.31	1.10	0.24	5.87	0.38	3.65	0.33	4.62
	0.16–6.25	0.24–5.82	0.16–0.55	3.43–7.05	0.21–1.93	3.01–4.48	0.14–0.75	2.48–7.88
Calprotectin (µg/g)	0.11	0.18	0.12	0.10	0.12	0.11	0.13	0.12
	0.10–0.14	0.16–0.32	0.09–0.13	0.07–0.14	0.07–0.14	0.07–0.12	0.09–0.14	0.09–0.12
IL-6 (>LLOD; *n* (%))	0 (0%)	7 (58%)	0 (0%)	0 (0%)	0 (0%)	1 (8%)	0 (0%)	0 (0%)
IL-8 (>LLOD; *n* (%))	0 (0%)	11 (92%)	0 (0%)	0 (0%)	0 (0%)	1 (8%)	0 (0%)	0 (0%)
IL-10 (>LLOD; *n* (%))	0 (0%)	0 (0%)	0 (0%)	0 (0%)	0 (0%)	0 (0%)	0 (0%)	0 (0%)
TNF-α (>LLOD; *n* (%))	0 (0%)	0 (0%)	0 (0%)	0 (0%)	0 (0%)	0 (0%)	0 (0%)	0 (0%)
CPV (positive; *n* (%))	0 (0%)	0 (0%)	0 (0%)	0 (0%)	0 (0%)	0 (0%)	0 (0%)	0 (0%)

Abbreviations: CPV, canine parvovirus; IL, interleukin; TNF-*α*, tumor necrosis factor alpha; LLOD, lower limit of detection.

**Table A2:** Hematological parameters (median and range, min–max) at baseline (Day 0) and after six weeks (Day 42) of placebo or probiotic supplementation in Labrador Retriever puppies.

	Placebo		HP1		HP2		HP1/2	
	Day 0	Day 42	Day 0	Day 42	Day 0	Day 42	Day 0	Day 42
RBC ($\times 10^{6}$/µL)	4.65	5.54	4.58	5.42	4.54	5.70	4.54	5.45
	4.32–4.93	4.97–5.83	4.39–4.81	5.20–5.71	4.34–4.87	5.21–5.90	3.80–4.85	5.23–5.63
Hemoglobin (g/dL)	9.9	11.2	9.9	11.0	9.7	11.7	9.7	11.3
	9.4–10.4	10.4–12.6	9.2–10.8	10.1–11.9	9.2–10.3	10.8–12.4	9.2–10.5	10.6–12.1
Hematocrit (%)	27.0	36.8	29.7	34.4	26.4	35.2	28.2	34.2
	25.4–31.3	35.1–38.4	27.0–33.3	33.6–35.9	25.8–27.3	31.1–37.0	25.1–32.3	30.8–36.4
MCV (fL)	65.3	64.0	66.3	62.0	64.5	62.0	67.2	62.4
	64.5–73.3	63.2–73.1	63.6–72.8	61.5–63.7	63.7–65.3	61.1–62.6	64.7–72.9	61.9–69.5
MCH (pg)	22.7	20.2	23.0	20.4	23.0	20.3	23.0	20.6
	22.5–23.8	19.4–23.1	21.5–23.7	20.1–21.0	22.8–23.3	19.5–20.7	22.0–24.3	20.1–21.1
MCHC (g/dL)	30.6	31.8	31.7	32.2	31.4	32.2	31.7	33.5
	28.7–34.1	30.5–34.8	30.3–32.5	30.9–32.9	29.3–32.0	31.1–33.6	29.6–33.5	31.8–34.0
RDW (%)	13.9	13.3	12.8	11.8	14.0	13.3	14.0	12.6
	11.8–15.0	12.3–13.9	12.3–13.9	11.4–12.7	13.4–15.6	12.1–14.4	12.8–15.2	11.9–14.2
HDW (g/dL)	2.33	2.18	2.35	2.05	2.36	2.07	2.49	2.14
	2.27–2.57	1.74–2.47	2.22–2.49	1.82–2.27	2.17–2.60	1.97–2.14	2.30–2.61	1.89–2.29
Reticulocytes (%)	5.01	3.47	5.69	1.71	5.17	2.66	5.31	1.77
	4.81–5.64	2.64–3.93	5.06–6.49	1.03–2.38	4.82–5.78	2.06–3.15	4.19–6.53	1.09–3.11
WBC ($\times 10^{3}$/µL)	19.44	20.04	17.09	14.36	18.76	18.29	17.28	17.02
	15.41–19.66	17.69–23.01	14.72–19.65	9.28–19.56	17.76–19.59	16.01–19.38	14.29–19.64	9.37–19.55
Neutrophils ($\times 10^{3}$/µL)	10.3	11.0	8.5	8.0	10.3	10.2	9.3	9.6
	8.4–11.0	10.1–14.6	6.3–10.8	5.7–10.9	9.7–10.7	9.3–10.7	6.0–11.7	5.8–11.1
Lymphocytes ($\times 10^{3}$/µL)	5.9	6.2	6.1	5.7	5.5	4.9	6.2	5.6
	5.3–6.8	4.6–6.6	5.5–6.5	3.9–6.7	4.6–5.9	4.1–6.4	3.7–8.2	1.9–6.5
Monocytes ($\times 10^{3}$/µL)	1.1	1.1	1.2	1.2	1.2	1.1	1.2	1.1
	1.0–1.4	0.7–1.4	1.0–1.4	0.9–1.4	0.9–1.4	0.9–1.4	1.0–1.6	0.8–1.4
Eosinophils ($\times 10^{3}$/µL)	0.3	0.3	0.4	0.4	0.4	0.5	0.3	0.5
	0.1–0.5	0.1–0.7	0.1–0.7	0.2–0.6	0.2–0.5	0.4–0.6	0.1–0.5	0.1–1.1
Basophils ($\times 10^{3}$/µL)	0.1	0.1	0.1	0.1	0.1	0.1	0.1	0.0
	0.0–0.4	0.0–0.2	0.0–0.1	0.0–0.1	0.0–0.1	0.0–0.1	0.0–0.1	0.0–0.1
Platelets ($\times 10^{3}$/µL)	315	544	363	506	291	532	312	494
	231–355	491–578	283–441	343–541	229–378	501–556	186–425	370–640
MPV (fL)	13.7	15.0	12.4	12.0	12.5	11.9	13.5	13.0
	11.5–15.5	11.4–15.9	12.0–13.9	11.5–13.6	11.3–13.8	11.5–13.7	12.0–14.7	11.6–15.0
PDW (%)	52.5	52.4	51.3	49.7	59.2	59.0	50.1	50.6
	42.5–58.5	43.4–58.8	49.8–54.1	47.5–55.1	52.7–64.7	52.4–65.0	42.0–57.3	39.9–63.1

Abbreviations: RBC, red blood cells; HGB, hemoglobin; HCT, hematocrit; MCV, mean corpuscular volume; MCH, mean corpuscular hemoglobin; MCHC, mean corpuscular hemoglobin concentration; RDW, red cell distribution width; HDW, hemoglobin distribution width; WBC, white blood cells; MPV, mean platelet volume; PDW, platelet distribution width.

**Table A3:** Blood biochemistry parameters (median and range, min–max) at baseline (Day 0) and after six weeks (Day 42) of placebo or probiotic supplementation in Labrador Retriever puppies.

	Placebo		HP1		HP2		HP1/2	
	Day 0	Day 42	Day 0	Day 42	Day 0	Day 42	Day 0	Day 42
Glucose (mg/dL)	72	72	102	105	102	109	81	111
	59–114	52–110	88–124	83–122	91–124	90–120	52–118	74–121
BUN (mg/dL)	17.0	9.2	16.8	9.0	17.1	8.3	18.3	9.2
	10.3–20.5	7.4–14.5	11.6–17.6	7.9–10.1	15.3–19.7	7.7–9.4	14.3–22.4	6.9–12.1
Uric acid (mg/dL)	0.9	0.9	0.9	0.9	0.9	0.9	0.8	0.6
	0.4–3.6	0.6–1.6	0.7–1.1	0.7–1.3	0.4–1.1	0.4–1.2	0.4–1.1	0.2–1.0
Creatinine (mg/dL)	1.2	1.1	1.3	1.1	1.2	1.0	1.3	1.0
	1.1–1.4	0.9–1.4	1.0–1.5	0.9–1.2	1.0–1.4	0.8–1.2	1.0–1.4	0.8–1.1
Total bilirubin (mg/dL)	0.84	0.80	0.89	0.72	0.83	0.69	0.90	0.45
	0.52–1.42	0.20–0.92	0.79–1.02	0.58–0.97	0.59–0.99	0.34–0.83	0.62–1.43	0.32–0.90
Conjugated bilirubin (mg/dL)	0.15	0.28	0.14	0.12	0.15	0.07	0.15	0.07
	0.11–0.34	0.01–0.31	0.04–0.25	0.04–0.21	0.06–0.23	0.03–0.19	0.05–0.26	0.01–0.26
Total cholesterol (mmol/L)	4.9	8.3	5.3	5.5	5.3	5.6	5.3	5.5
	4.4–5.8	5.1–9.1	4.7–5.7	5.1–5.9	4.9–5.9	5.4–5.8	4.7–5.6	4.9–8.4
Potassium (mmol/L)	5.3	4.8	6.1	5.7	5.4	5.3	6.3	5.4
	4.1–6.8	3.3–5.4	5.4–6.4	5.1–6.0	4.4–6.1	4.7–6.5	4.3–7.1	4.8–6.1
Chloride (mmol/L)	111	106	102	98	105	106	108	106
	101–118	92–109	94–109	84–106	100–115	93–109	105–115	102–107
Sodium (mmol/L)	143	142	143	141	143	142	141	144
	141–147	130–147	133–150	131–145	137–147	133–149	141–145	140–149
Calcium (mmol/L)	10.9	10.7	11.2	11.7	11.0	11.1	11.6	11.8
	10.4–12.0	10.0–12.0	10.1–11.4	11.3–11.9	10.2–11.9	10.9–12.0	10.9–12.0	11.4–12.1
Phosphorus (mmol/L)	10.1	8.7	9.6	8.8	10.3	8.7	9.9	8.9
	8.4–11.1	8.2–9.6	9.1–10.3	8.4–9.2	9.3–10.7	8.4–9.3	9.3–10.6	8.7–9.6
ALP (U/L)	186	153	165	157	157	151	158	161
	117–254	101–195	143–173	132–167	121–207	131–187	127–216	142–229
AST (U/L)	37	42	37	39	37	39	38	36
	31–45	37–74	32–39	34–43	27–40	33–44	33–47	31–44
ALT (U/L)	22	27	23	29	26	28	21	25
	14–25	21–44	19–26	26–31	21–32	26–34	14–25	18–30
GGT (U/L)	5	6	4	4	5	5	5	5
	3–6	2–8	4–6	3–6	3–6	4–6	4–7	2–7
Total LDH (U/L)	392	420	353	375	190	207	359	308
	198–523	123–712	325–369	316–391	154–291	124–293	219–411	173–412
CPK (U/L)	501	617	445	434	386	384	395	433
	332–629	307–748	369–603	349–579	358–592	306–584	328–633	252–641
Amylase (U/L)	828	951	533	526	454	499	698	670
	420–1067	596–1247	425–969	442–978	403–892	400–919	435–1002	565–1010
Lipase (U/L)	46	50	40	32	45	37	52	33
	29–76	25–65	33–49	29–47	37–59	31–51	34–75	19–61
Total proteins (g/dL)	4.40	4.65	4.90	4.65	4.90	4.70	4.55	4.80
	4.0–5.6	3.9–6.1	4.0–5.3	4.4–4.8	4.6–5.2	4.0–4.9	4.0–5.3	4.0–5.2
Albumins (g/dL)	2.30	2.55	2.60	2.40	2.55	2.55	2.30	2.45
	1.7–2.9	2.0–3.4	2.1–2.9	2.0–2.7	2.4–2.9	2.0–2.6	1.7–2.9	2.0–2.76
Globulins (g/dL)	2.20	2.10	2.30	2.40	2.40	2.10	2.25	2.40
	1.9–2.7	1.8–2.8	1.9–2.6	2.0–2.4	2.1–2.4	2.0–2.4	1.9–2.8	2.0–2.9
A/G ratio	1.05	1.18	1.20	1.00	1.08	1.21	1.03	1.00
	0.74–1.23	1.00–1.28	0.92–1.35	0.83–1.35	1.00–1.26	1.00–1.24	0.74–1.23	0.72–1.35

Abbreviations: A/G, albumin: globulin ratio; BUN, blood urea nitrogen; ALP, alkaline phosphatase; AST, aspartate aminotransferase; ALT, alanine aminotransferase; GGT, gamma-glutamyl transferase; LDH, lactate dehydrogenase; CPK, creatine phosphokinase.

**Table A4:** Additional blood and immunological parameters (median and range, min–max) at baseline (Day 0) and after six weeks (Day 42) of placebo or probiotic supplementation in Labrador Retriever puppies.

	Placebo		HP1		HP2		HP1/2	
	Day 0	Day 42	Day 0	Day 42	Day 0	Day 42	Day 0	Day 42
Cortisol (µg/dL)	2.62	4.37	2.88	2.19	2.98	2.19	2.97	2.41
	1.97–3.04	3.68–4.98	2.30–3.29	1.98–3.21	2.41–3.45	2.02–2.42	1.99–3.42	1.87–2.61
IL-8 (ng/mL)	2.85	4.36	2.86	2.54	3.20	2.58	3.32	2.49
	1.29–3.25	3.28–6.63	2.05–4.01	2.06–2.90	2.76–4.00	2.08–2.90	2.56–4.15	2.32–3.16
TNF-α (pg/mL)	2.72	3.89	3.67	3.57	3.63	3.12	2.82	2.30
	1.67–3.88	2.79–8.63	2.94–3.84	3.01–4.07	2.27–3.97	1.91–4.21	1.97–4.32	1.65–4.03
IL-10 (>LLOD; *n* (%))	0 (0%)	4 (33%)	0 (0%)	0 (0%)	0 (0%)	1 (8%)	0 (0%)	0 (0%)
CPV titer	NA	4	NA	5	NA	5	NA	5
		3–4		4–6		4–6		4–6
CDV titer	NA	3	NA	4	NA	4	NA	5
		2–3		3–5		3–5		3–5
cPLI (>40 µg/L; *n* (%))	0 (0%)	0 (0%)	0 (0%)	0 (0%)	0 (0%)	0 (0%)	0 (0%)	0 (0%)

Abbreviations: cPLI, canine pancreatic specific lipase; CPV, canine parvovirus; CDV, canine distemper virus; IL, interleukin; LLOD, lower limit of detection; NA, not analyzed; TNF-*α*, tumor necrosis factor alpha.

**Table A5:** Summary of Kruskal–Wallis tests and Dunn’s post-hoc pairwise comparisons versus placebo for hematological, biochemical, endocrine, and fecal parameters in Labrador Retriever puppies after six weeks (Day 42) of probiotic or placebo supplementation.

					HP1 vs. Placebo		HP2 vs. Placebo		HP1/2 vs. Placebo	
Parameter	$\chi^{2}$	df	p	$\varepsilon^{2}$	r	p	r	p	r	p
**Hematology**										
RBC	10.564	3	0.014	0.225	0.083	1.000	0.463	0.140	0.123	1.000
Hemoglobin	7.491	3	0.058	0.159	0.171	1.000	0.373	0.407	0.025	1.000
Hematocrit	20.407	3	<0.001	0.434	0.736	0.002	0.404	0.288	0.832	<0.001
MCV	26.847	3	<0.001	0.571	0.805	<0.001	0.995	<0.001	0.560	0.036
MCH	4.501	3	0.212	0.096	0.126	1.000	0.045	1.000	0.350	0.520
MCHC	9.253	3	0.026	0.197	0.025	1.000	0.062	1.000	0.475	0.120
RDW	18.815	3	<0.001	0.400	0.772	<0.001	0.012	1.000	0.289	0.940
HDW	3.897	3	0.273	0.083	0.250	1.000	0.332	0.623	0.025	1.000
Reticulocytes	28.239	3	<0.001	0.601	0.996	<0.001	0.496	0.091	0.848	<0.001
WBC	11.169	3	0.011	0.238	0.583	0.026	0.542	0.048	0.542	0.048
Neutrophils	14.624	3	0.002	0.311	0.743	0.002	0.500	0.085	0.567	0.033
Lymphocytes	7.109	3	0.069	0.151	0.145	1.000	0.514	0.071	0.319	0.709
Monocytes	0.820	3	0.845	0.017	0.122	1.000	0.169	1.000	0.047	1.000
Eosinophils	6.749	3	0.080	0.144	0.210	1.000	0.526	0.059	0.262	1.000
Basophils	3.445	3	0.328	0.073	0.112	1.000	0.112	1.000	0.216	1.000
Platelets	9.005	3	0.029	0.192	0.549	0.043	0.118	1.000	0.387	0.348
MPV	20.537	3	<0.001	0.437	0.796	<0.001	0.793	<0.001	0.432	0.205
PDW	15.287	3	0.002	0.325	0.228	1.000	0.524	0.062	0.070	1.000
**Blood biochemistry**										
Glucose	20.423	3	<0.001	0.435	0.688	0.005	0.774	<0.001	0.783	<0.001
BUN	3.463	3	0.326	0.074	0.077	1.000	0.329	0.641	0.004	1.000
Uric acid	9.582	3	0.022	0.204	0.058	1.000	0.142	1.000	0.516	0.069
Creatinine	5.340	3	0.149	0.114	0.140	1.000	0.416	0.250	0.356	0.489
Total Bilirubin	9.534	3	0.023	0.203	0.086	1.000	0.332	0.622	0.570	0.031
Conjugated Bilirubin	10.724	3	0.013	0.228	0.245	1.000	0.587	0.024	0.534	0.054
Total Cholesterol	18.678	3	<0.001	0.397	0.798	<0.001	0.585	0.025	0.714	0.003
$K^{+}$	16.780	3	<0.001	0.357	0.803	<0.001	0.572	0.031	0.560	0.037
${\mathrm{Cl}}^{-}$	13.701	3	0.003	0.292	0.552	0.041	0.006	1.000	0.150	1.000
${\mathrm{Na}}^{+}$	5.868	3	0.118	0.125	0.181	1.000	0.064	1.000	0.308	0.791
${\mathrm{Ca}}^{2+}$	18.644	3	<0.001	0.397	0.610	0.017	0.134	1.000	0.742	0.002
P	4.257	3	0.235	0.091	0.022	1.000	0.108	1.000	0.296	0.883
ALP	5.501	3	0.139	0.117	0.083	1.000	0.211	1.000	0.259	1.000
AST	10.721	3	0.013	0.228	0.387	0.347	0.335	0.606	0.665	0.007
ALT	8.684	3	0.034	0.185	0.193	1.000	0.205	1.000	0.321	0.697
GGT	3.860	3	0.277	0.082	0.355	0.491	0.058	1.000	0.239	1.000
LDH	21.516	3	<0.001	0.458	0.308	0.787	0.921	<0.001	0.514	0.070
CPK	14.727	3	0.002	0.313	0.527	0.059	0.722	0.002	0.609	0.017
Amylase	19.333	3	<0.001	0.411	0.661	0.007	0.810	<0.001	0.286	0.969
Lipase	7.575	3	0.056	0.161	0.439	0.190	0.088	1.000	0.434	0.201
Total Protein	2.928	3	0.403	0.062	0.111	1.000	0.126	1.000	0.183	1.000
Albumin	6.006	3	0.111	0.128	0.435	0.200	0.005	1.000	0.120	1.000
Globulin	6.000	3	0.112	0.128	0.195	1.000	0.192	1.000	0.259	1.000
A:G ratio	14.559	3	0.002	0.310	0.566	0.033	0.064	1.000	0.457	0.152
**Extra blood parameters**										
Cortisol	29.336	3	<0.001	0.624	0.917	<0.001	0.993	<0.001	0.662	0.007
IL-8	26.594	3	<0.001	0.566	0.857	<0.001	0.819	<0.001	0.896	<0.001
TNF-α	17.316	3	<0.001	0.368	0.180	1.000	0.452	0.160	0.796	<0.001
CPV titer	28.101	3	<0.001	0.598	0.856	<0.001	0.804	<0.001	0.961	<0.001
CDV titer	26.053	3	<0.001	0.554	0.773	<0.001	0.723	0.002	0.973	<0.001
**Fecal parameters**										
Fecal Score	35.967	3	<0.001	0.765	0.965	<0.001	1.000	<0.001	0.892	<0.001
Fecal dry matter	26.708	3	<0.001	0.568	0.900	<0.001	0.811	<0.001	0.864	<0.001
Butyrate	22.400	3	<0.001	0.477	0.801	<0.001	0.470	0.127	0.860	<0.001
Acetate	24.669	3	<0.001	0.525	0.644	0.010	0.162	1.000	0.890	<0.001
Propionate	26.629	3	<0.001	0.567	0.978	<0.001	0.333	0.624	0.721	0.003
Calprotectin	30.372	3	<0.001	0.646	1.000	<0.001	0.936	<0.001	0.652	0.008

Kruskal–Wallis test results are reported with *χ*^2^, degrees of freedom (df), and effect size (*ε*^2^). Pairwise Dunn’s tests versus placebo are reported with effect size (r) and Bonferroni-adjusted *p*-values applied per parameter.
